# Genetic Diversity of *Plasmodium falciparum* Merozoite Surface Protein 1 and 2 Genes and Their Allele Association With Malaria Severity, Seasons, and Sex in Bamenda, North West Cameroon

**DOI:** 10.1155/cjid/8378496

**Published:** 2026-01-08

**Authors:** Che Roland Achungu, Damian Nota Anong, Kamena Faustin, Robert Adamu Shey, Cevie Jesenta Tabe, Chi Achille Djouosseu

**Affiliations:** ^1^ Department of Microbiology and Parasitology, University of Buea, Buea, South West Region, Cameroon, ubuea.cm; ^2^ Laboratory of Infectious Disease Research, Bamenda, North West Region, Cameroon; ^3^ Florence Nightingale Higher Institute of Health and Biomedical Sciences, Bamenda, North West Region, Cameroon; ^4^ Department of Biological Sciences, The University of Bamenda, Bamenda, North West Region, Cameroon, unibda.net; ^5^ Department of Biochemistry and Molecular Biology, University of Buea, Buea, South West Region, Cameroon, ubuea.cm; ^6^ Department of Biochemistry, The University of Bamenda, Bamenda, North West Region, Cameroon, unibda.net

## Abstract

Although the number of yearly occurrences of malaria has significantly decreased in recent years, malaria remains one of the major public health issues in Cameroon. A deeper understanding of how the various alleles of the malaria parasite’s genes move in the field between the two sexes and between seasons, as well as knowledge of malaria parasite genetic diversity in different malaria‐endemic locations, would be necessary for the development of a successful malaria elimination strategy. This study aimed to evaluate the genetic diversity of the Msp1 and Msp2 genes of *Plasmodium falciparum* in Bamenda, North West Region of Cameroon, identify the alleles of the Msp1 and Msp2 genes that are associated with severe malaria, and determine how the alleles of the Msp1 and Msp2 genes are distributed between sexes and seasons. Blood samples were collected on Whatman’s filter paper from those who had malaria caused by *P. falciparum* as determined by microscopy and rapid diagnostic tests. DNA from *P. falciparum* was isolated using the Chelex technique. Using nested PCR, the Msp1 Block 2 and Msp2 Block 3 genes were genotyped. The Msp1 Block 2 gene was genotyped in a total of 281 samples. For Msp1 Block 2 genes, all the family‐specific alleles were present (RO33, K1, and MAD20). At 44.7% frequency, RO33 was the most prevalent allele, while K1 was linked to severe malaria. The Msp2 Block 3 gene was genotyped in a total of 194 samples. For Msp2 Block 3 genes, all of the family‐specific alleles were present (3D7 and FC27). With 56.7%, 3D7 was the most prevalent. There was an association between the Msp2 Block 3 gene alleles and sex (χ^2^ = 8.856, *p* = 0.012). No Msp1 Block 2 or Msp2 Block 3 allele was more prevalent in any specific season. These results would be useful in assessing the efficacy of malaria prevention measures implemented in Bamenda as well as in selecting and designing malaria prevention measures that are suitable for implementation in Bamenda, North West Region of Cameroon.

## 1. Introduction

Given its high global morbidity and mortality rates, malaria continues to be a major public health concern. Malaria is a parasite disease spread by female Anopheles mosquitoes. *Plasmodium falciparum, Plasmodium vivax, Plasmodium malariae, Plasmodium ovale,* and *Plasmodium knowlesi* are the five protozoa that cause malaria in humans [1]. *P. vivax* is the leading cause of malaria worldwide, whereas *P. falciparum,* which causes more than 90% of global malaria death, continues to be the single most significant hazard to public health on a global level [1]. The estimated number of malaria cases and deaths globally in 2023 was 263 million and 597,000, respectively [2]. Study had shown that in some malaria‐endemic areas, children have a primary malaria attack during their first year of life, while toddlers and juveniles have already developed resistance against severe disease but still experience a few clinical episodes. Adolescents and adults, in contrast, are often clinically immune to severe malaria despite continuous exposure to the malaria parasite [3]. Malaria is endemic in Cameroon and has a devastating impact on public health and welfare. There has been increased scale‐up of vector control interventions and the use of Artemisinin combination therapies (ACTs), which have significantly reduced the prevalence of malaria across Cameroon from 41% in the year 2000 to 24% in the year 2017 [4]. However, the malaria situation in Cameroon, like many other countries in sub‐Saharan Africa, is not yet under control because of the development and potential spread of artemisinin resistance in the malaria parasite, insecticide resistance in the mosquito vector, the insufficient protection provided by the RTS, S vaccine, and insufficient funds for malaria control and elimination [5].

Among human malaria parasites, *P. falciparum* is characterized by high genetic diversity in several populations of malaria parasite isolates [6]. This high genetic diversity is an obstacle to malaria control and elimination efforts. Thus, understanding the genetic diversity of *P. falciparum* could support the current malaria control and elimination efforts. Polymerase Chain Reaction (PCR)‐based genotyping of *P. falciparum’*s genes with high genetic diversity, such as Merozoite Surface Protein 1 (Msp1) and Merozoite Surface Protein 2 (Msp2), is the most popular and commonly used method for determining the genetic diversity of *P. falciparum* [7, 8]. The Msp1 gene is located on Chromosome 9 and contains 17 blocks of sequences [9]. Block 2 represents an exception to all the blocks with the third allele (RO33) in addition to KI and MAD20 [9]. The Msp2 gene is located on Chromosome 2 and composed of five blocks and Block 3 is the most polymorphic [10]. The Msp2 Block 3 alleles are grouped in two allelic families, FC27 and ICI/3D7 [11]. Msp1 and Msp2 genes exhibit high diversity and hence play a key role in the identification of genetically distinct *P. falciparum* subpopulations [11] and also in the study of malaria transmission dynamics across seasons [12]. Msp1 mixed alleles of K1 and RO33 have been found to be strongly associated with severe malaria [13]. Additionally, the FC27 allele of Msp2 has been linked to severe malaria [14]. Msp1 and Msp2 genes encode the Msp1 and Msp2, respectively. Msp1 and Msp2 are proteins expressed on the surface of the merozoite and are involved in erythrocyte invasion [15] and are targeted by the host immune system [15]. Msp1 and Msp2 are among the leading malaria vaccine candidate antigens [16]. The development of effective malaria elimination strategies would require a better understanding of how the different alleles of the *P. falciparum’s* Msp1 and Msp2 genes are linked to malaria severity and how the alleles circulate in the field between sexes and between seasons.

In Cameroon, genetic diversity of *P. falciparum* Msp1 and Msp2 genes circulating in other regions of Cameroon has been extensively characterized [8, 17–19]. However, there has been no research on the genetic diversity of *P. falciparum* genes in the North West Region of Cameroon. This study aimed to evaluate the genetic diversity of the Msp1 and Msp2 genes of *P. falciparum* in Bamenda, North West Region of Cameroon, identify alleles of the Msp1 and Msp2 genes that are associated with severe malaria, and determine how the alleles of the Msp1 and Msp2 genes are distributed between sexes and seasons. These data would be useful in assessing the efficacy of malaria prevention measures implemented in Bamenda as well as in selecting and designing malaria prevention measures that are suitable for implementation in Bamenda, North West Region of Cameroon.

## 2. Methods

### 2.1. Study Area

The research was conducted from June 2019 to February 2020 in Bamenda, which is the capital of Cameroon’s North West Region. Bamenda is located between latitude 6° N and longitude 10.1°E with an average altitude of 1472 m above sea level. The climate is classified as a tropical monsoon with two seasons: dry season from November to March and rainy season from April to October. The annual rainfall is 2300 mm. Bamenda, which has a population of about 900,000, is 366 km northwest of Yaounde, the capital of Cameroon. Because it is an urban region with individuals from several ethnic groups, its population is genetically varied. The spread of malaria is ongoing. The frequency of malaria rises in the wet season and declines in the dry. Bamenda is situated in a highland area (greater than 1000°m above sea level). It is an area of low malaria transmission, *Anopheles gambiae* is the main vector species, and *P. falciparum* is the main parasite species [4].

### 2.2. Sample Collection and Processing

166 boys and 156 girls, aged 0–17 years, who are all from the Semi‐Bantu ethnic group with no history of immunosuppressive diseases and displayed malarial symptoms and clinical indications had their venous blood samples taken. Before taking venous blood samples, the participant’s axillary temperature was recorded using a digital thermometer. A total of 322 blood samples were collected. The URIT‐12 Hemoglobin Meter was used to determine the participants’ hemoglobin levels from each participant’s blood sample. The blood samples were subjected to rapid diagnostic test for malaria and malaria microscopy. For each participant’s blood sample, the malaria parasite density was calculated using the following formula:

(1) The number of asexual parasites counted×8000 leucocytes/μL of bloodThe number of white blood cells counted.

Blood samples positive for *P. falciparum* were spotted on Whatman filter papers packaged individually in zip‐locked bags and appropriately labeled. The samples were examined at the University of Buea’s Molecular Parasitology Laboratory.

### 2.3. Malaria Parasite DNA Extraction

*P. falciparum* genomic DNA was extracted using the Chelex method of DNA extraction as described previously [20]. Agarose gel electrophoresis was used to confirm the presence of genomic DNA. The extracted DNA was kept at −20°C until it was needed.

### 2.4. Genotyping of *P. falciparum* Msp1 and Msp2 Genes

This was done using a Nested PCR approach as described previously [21].

#### 2.4.1. Primary PCR

The PCRs were performed on a Gene AMP PCR 9700 Applied Biosystem Machine. The PCR mixture contained 10 μL of One Taq Hot Start Quick‐Load (from New England Biolabs), 0.8 µL of forward primers, 0.8 μL of reverse primers, 1 μL of DNA template from the extracted DNA, and 7.4 µL of nuclease‐free water, making a total volume of 20 μL. The primers used were primers that amplify the complete *P. falciparum* Msp1 and Msp2 genes. The PCR was programmed for 25 cycles [21].

#### 2.4.2. Secondary PCR

The PCRs were performed on a Gene AMP PCR 9700 Applied Biosystem Machine. The PCR mixture contained 10 μL of One Taq Hot Start Quick‐Load (from New England Biolabs), 0.8 µL of forward primers, 0.8 μL of reverse primers, 1 μL of DNA template obtained from the product of the first round of PCR, and 7.4 µL of nuclease‐free water, making a total volume of 20 μL. The primers used for Msp1 gene were primers that amplified the different alleles of Msp1 Block 2 gene, whereas those for Msp2 were primers that amplified the different alleles of Msp2 Block 3 gene. The PCR was programmed for 30 cycles [21].

PCRs were performed as follows: initial denaturation at 95°C for 5 min, followed by 25 cycles (first round) or 30 cycles (second round) of denaturation at 94°C for 1 min, annealing at 58°C for 2 min, and extension at 72°C for 2 min. Final extension was carried out at 72°C for 5 min and held at 5°C. The negative control’s PCR settings were the same as those used in the experiment, but it did not contain any of the genomic DNA that was extracted from *P. falciparum*.

The primers used for genotyping of *P. falciparum* Msp1 and Msp2 genes are given in Tables 1 and 2, respectively.

**Table 1:** Primers used to genotype *P. falciparum* Msp1 Block 2 gene.

Target	Primer name	Sequence 5′‐3′
MSP1 First round	MI‐OF	CTA​GAA​GCT​TTA​GAA​GAT​GCA​GTA​TTG
	MI‐OR	CTT​AAA​TAG​TAT​TCT​AAT​TCA​AGT​GGA​TCA

KI‐type allele	MI‐KF	AAA​TGA​AGA​AGA​AAT​TAC​TAC​AAA​AGG​TGC
	MI‐KR	GCT​TGC​ATC​AGC​TGG​AGG​GCT​TGC​ACC​AGA

MAD20‐type allele	MI‐MF	AAA​TGA​AGG​AAC​AAG​TGG​AAC​AGC​TGT​TAC
	MI‐MR	ATC​TGA​AGG​ATT​TGT​ACG​TCT​TGA​ATT​ACC

RO33‐type allele	MI‐RF	TAA​AGG​ATG​GAG​CAA​ATA​CTC​AAG​TTG​TTG
	MI‐RR	CAT​CTG​AAG​GAT​TTG​CAG​CAC​CTG​GAG​ATC

**Table 2:** The primers used to genotype *P. falciparum* Msp2 Block 3 gene.

Target	Primers	Sequence 5′‐3′
MSP2	M2‐OF	ATG​AAG​GTA​ATT​AAA​ACA​TTG​TCT​ATT​ATA
	M2‐OR	ATA​TGG​CAA​AAG​ATA​AAA​CAA​GTG​TTG​CTG

FC 27‐type allele	M2‐FCF	GCA​AAT​GAA​GGT​TCT​AAT​ACT​AAT​AG
	M2‐FCR	GCT​TTG​GGT​CCT​TCT​TCA​GTT​GAT​TC

IC/3D7‐type allele	M2‐ICF	GCA​GAA​AGT​AAG​CCT​TCT​ACT​GGT​GCT
	M2‐ICR	GAT​TTG​TTT​CGG​CAT​TAT​TAT​GA

### 2.5. Visualization of Msp1 and Msp2 PCR Products Using Agarose Electrophoresis

**Figure 1:**
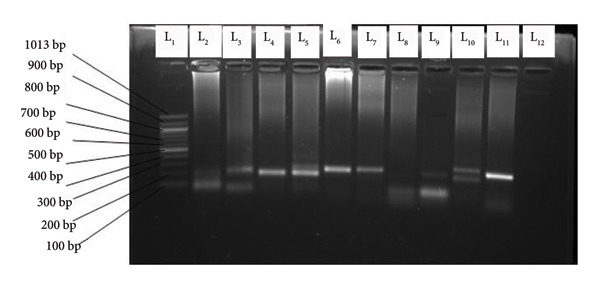
Variants of K1: L1 = molecular weight marker (1000 bp), L2 = 100 bp, L3 = 250 bp and 100 bp, L4 = 180 bp, L5 = 180 bp, L6 = 200 bp, L7 = 200 bp, L9 = 200 bp and 180 bp, L10 = 200 bp and 200 bp, L11 = 180 bp, L12 = negative control.

**Figure 2:**
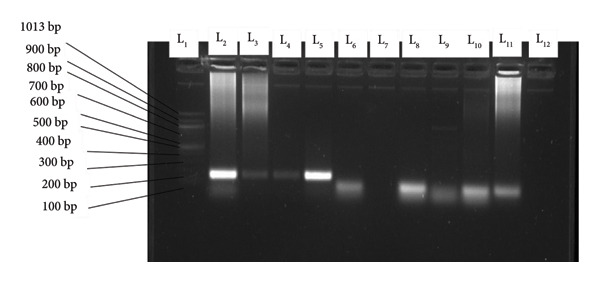
Variants of RO33 and MAD20: L1 = MW marker (1000 bp), L2–L5 = R033, all 180 bp, L6, L8, L9, L10, L11 = MAD20, all 100 bp and L12 = negative control.

**Figure 3:**
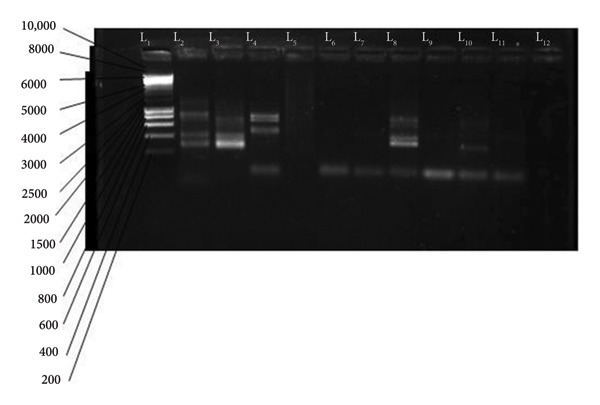
Variants of 3D7: L1 = MW marker (1 Kb), L1 = 800, 400, and 300 bp, L2 = 600, 400, and 300 bp, L4 = 600, 500, and 100 bp, L6 = 100 bp, L7 = 100 bp, *L*8 = 100 and 200 bp, *L*9 = 100 bp, L10 = 200 and 100 bp, L11 = 100 bp, and L12 = negative control.

**Figure 4:**
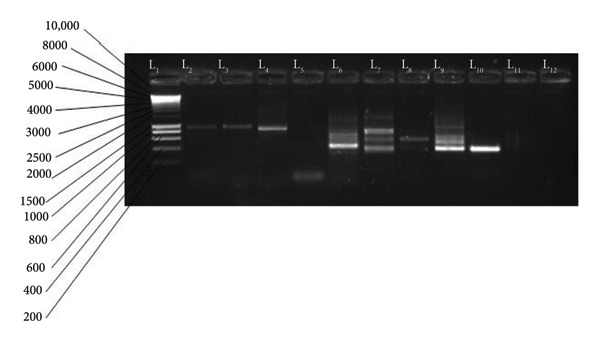
FC27 variants: L1 = MW marker (1 Kb), L2–L3 = 1000 bp, L4 = 800 bp, L5 = 400 bp, L6 = 400 bp, L7 = 600, 500, and 400 bp, L8 = 500 bp, L9–L10 = 400 bp, and L12 = negative.

The amplified products of the secondary PCR were visualized using 1.5% agarose gel following electrophoresis. The agarose gel contained 1.5% agarose mixed with 1 µL of ethidium bromide. The electrophoresis ran for 40 min at 100 V. The migration occurred in a gel fill with 1 X TBA buffer. The molecular weight marker (1000 bp for Msp1 or 10000 bp for Msp2) was loaded in the first well. The migrated DNA products were visualized using the gel documentation system (Bio‐Rad, USA). The migrated amplified DNA fragments for the different alleles for Msp1: KI, are shown in Figure 1, those of MAD 20 and RO33 in Figure 2, and for Msp2: 3D7 in Figure 3 and FC27 in Figure 4.

### 2.6. Analysis

After laboratory analysis, participant data were recorded on laboratory worksheets. A database created with Microsoft Excel was filled out with survey responses and lab findings. A sample was deemed to belong to a certain allelic family for the Msp1 Block 2 gene or the Msp2 Block 3 gene if, after the second round of PCR using the family‐specific primers, at least one band appeared. The frequency of each allele was calculated by dividing the proportion of fragments allocated to each allele by the total number of fragments found at either the Msp1 Block 2 gene locus for Msp1 Block 2 alleles or the Msp2 Block 3 gene locus for Msp2 Block 3 alleles. A sample was considered to have a mixed infection if it had two or three distinct alleles of the Msp1 Block 2 or Msp2 Block 3 genes. The SPSS version 26 program was used to analyze all the data, which were imported into Excel. Pearson’s *χ* ^2^ test was used to determine the importance of variations in the allele distributions of the Msp1 Block 2 and Msp2 Block 3 genes. To determine if there was a significant difference between the mean parasite density of the K1 samples and a high mean parasite density of 10,000 trophozoites/μL, an uptailed *Z*‐test was utilized. A difference was deemed statistically significant if the *p*‐value was less than 0.05.

## 3. Results

### 3.1. Demographic, Parasitological, and Clinical Characteristics of Study Population

All of the participants were children from the Semi‐Bantu ethnic group, ranging in age from 0 to 17 years. A total of 322 children were enrolled in the study. The children’s average body temperature was 38.5°C, their average hemoglobin level was 11.1 g/dL, and their average parasite density for malaria was 16,503 trophozoites/μL (Table 3).

**Table 3:** Demographic, parasitological, and clinical characteristics of study population.

Number of participants	281 participants
Ethnicity	281 Semi‐Bantu children
Sex	Male = 166 participants Female = 156 participants
Age ranges	0–5 years = 180 participants 6–11 years = 96 participants 12–17 years = 46 participants
Period of study	Dry season = 116 participants Rainy season = 206
Bed net usage	Number of participants sleeping under bed net = 153 Number of participants who do not sleep under bed net = 169
Malaria parasite species found	322 all *P. falciparum*
Axillary temperature	Mean temperature of participants = 38.53°C, SD = 1.03, maximum value = 43°C, minimum value = 35.5°C
Hemoglobin level	Mean hemoglobin level = 11.1 g/dL Maximum value = 16 g/dL, minimum value = 4.7 g/dL
Parasite density	Mean parasite density = 16503 trophozoites/μL minimum = 40 trophozoites/μL, maximum = 400,000 trophozoites/μL

### 3.2. Identification of Different Allelic Types of *P. falciparum* Msp1 Block 2 Gene in the Study Area

**Figure 5:**
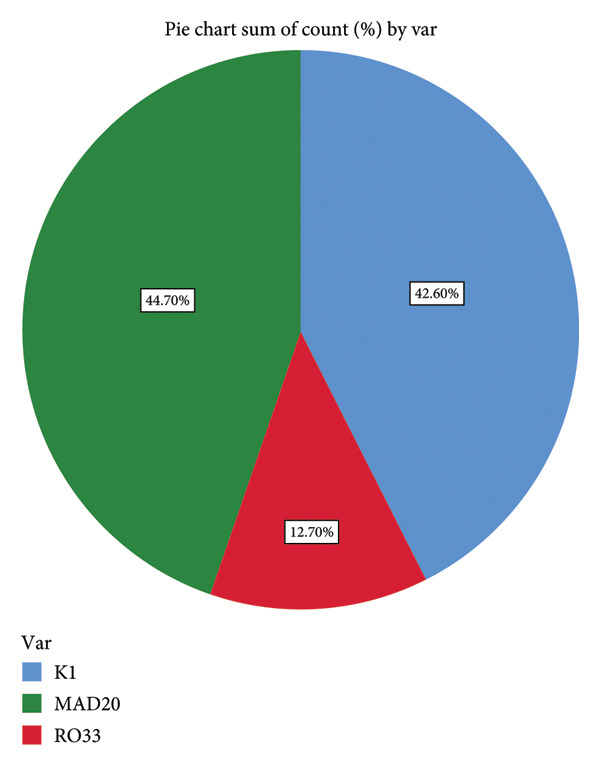
Frequency of *P. falciparum* Msp1 Block 2 alleles in Bamenda.

For the Msp1 Block 2 gene, a total of 281 samples were genotyped. The outcome demonstrates the presence of all three MSP1 Block 2 alleles (K1, MAD20, and RO33) in the study area. With a percentage of 44.7%, RO33 recorded the greatest allelic frequency (Figure 5). The results indicate that there is a high genetic variation within K1 allelic family with variants ranging from 100 to 300 bp (100 bp found in 25 samples, 180 bp found in 10 samples, 200 bp found in 74 samples, 250 bp found in 18 samples, and 300 bp found in 3 samples) as compared to the other allelic families. MAD20 allelic family shows limited genetic diversity with variants ranging from 100 to 250 bp (100 bp found in 10 samples, 150 bp found in 2 samples, 200 bp found in 24 samples, and 250 bp found in 1 sample), while there was no genetic variation within the R033 allelic family with all 159 samples containing 180 bp. In both K1 and MAD20, the 200 bp variant was the most prevalent.

### 3.3. Identification of Different Allelic Types of *P. falciparum* Msp2 Block 3 Gene in the Study Area

**Figure 6:**
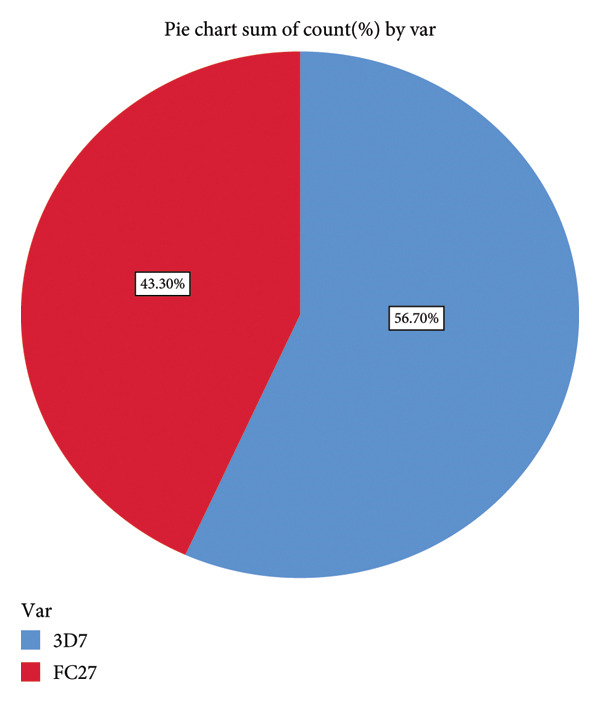
Frequency of *P. falciparum* Msp2 Block 3 alleles in Bamenda.

A total of 194 samples were successfully amplified for the *P. falciparum* Msp2 Block 3 gene. 161 samples gave 214 fragments of 3D7 that range from 100 to 1000 bp (95 samples with 100 bp, 60 samples with 200 bp, 3 samples with 300 bp, 39 samples with 400 bp, 5 samples with 500 bp, 8 samples with 600 bp, 3 samples with 800 bp, and 1 sample with 1000 bp). While 90 samples gave 168 fragments of FC27 that range from 100 to 1200 bp (2 samples with 100 bp, 10 samples with 200 bp, 19 samples with 300 bp, 89 samples with 400 bp, 2 samples with 500 bp, 13 samples with 600 bp, 14 samples with 700 bp, 3 samples with 800 bp, 10 samples with 1000 bp, and 6 samples with 1200 bp). The result suggests that the study area has all two alleles of the Msp2 Block 3 genes, and the most predominant allele is 3D7 (Figure 6). *P. falciparum* Msp2 Block 3 gene is usually studied as a marker of genetic diversity of natural populations of Plasmodium species. *P. falciparum* Msp2 Block 3 can be used to assess malaria control strategies in the field. Mono‐infection (infections with either 3D7 or FC27) were 36.6% for 3D7 and 17% for FC27, while mixed infections were 46.6%.

### 3.4. Association of Msp1 and Msp2 Alleles With Malaria Severity in Bamenda

For Msp1 Block 2, the results indicate that mono‐infection with the K1 allele causes the study participants to have high mean malaria parasite densities, high mean temperatures, and low mean hemoglobin levels. Low mean malaria parasite density, low mean temperature, and high mean hemoglobin levels are seen among study participants with mono‐infection carrying the RO33 allele (Table 4). Parasite density of K1 samples was compared to 10,993 trophozoites/μL (corresponding to severe malaria in Semi‐Bantu children as determined by [22]) using one sample upper‐tailed *Z*‐test with *p* ≤ 0.001, indicating that K1 allele might be responsible for malaria disease severity in Bamenda. For the Msp2 block gene, the findings show that individuals with the FC27 allele have slightly higher parasite densities than those with the 3D7 alleles, and that mixed infections (3D7_ FC27) have a slightly higher mean parasite density compared to mono‐infections (3D7 or FC27), but the differences observed were not statistically significant.

**Table 4:** Association of *P. falciparum* Msp1 Block 2 gene with respect to mean parasite density, mean temperature, and mean hemoglobin levels among study participants.

Msp1 alleles	Mean parasite density (trophozoites/μL)	Mean temperature (°C)	Mean hemoglobin level (g/dL)
RO33	5801	38.5	11.3
K1	33,900	38.6	11.1
MAD20	9729	38.6	11.2
RO33 and K1	27,079	38.1	11.6
RO33 and MAD20	800	38.6	11.5
K1 and MAD20	25,524	38.8	11.3
K1, MAD 20 and RO33	66,000	40.1	9.1

### 3.5. Association of Msp1 and Msp2 Alleles With Seasons in Bamenda

**Figure 7:**
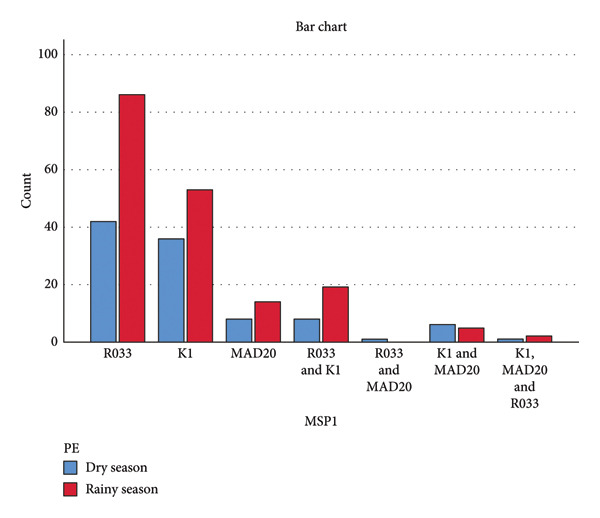
Distribution of Msp1 Block 2 alleles with respect to seasonality (Pearson *χ* ^2^ = 5.206, *p* = 0.518, linear by linear association *p* = 0.344) PE represents period of enrollment.

**Figure 8:**
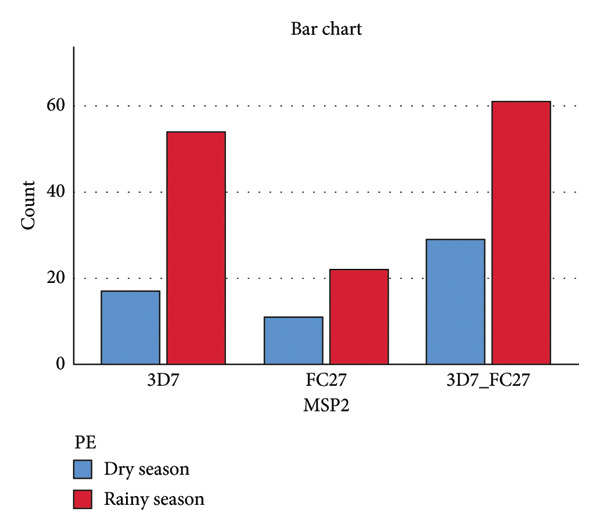
Distribution of Msp2 Block 3 alleles with respect to seasonality (Pearson *χ* ^2^ = 1.610, *p* = 0.447) PE represents period of enrollment.

The result shows that seasonality has a little impact on the distribution of Msp1 Block 2 and Msp2 Block 3 alleles in the study area since individuals might contract the same alleles during both wet and dry seasons. No Msp1 Block 2 allele (Figure 7) or Msp2 Block 3 allele (Figure 8) was associated with a particular season.

### 3.6. Association of Msp1 and Msp2 Alleles With Sex in Bamenda

**Figure 9:**
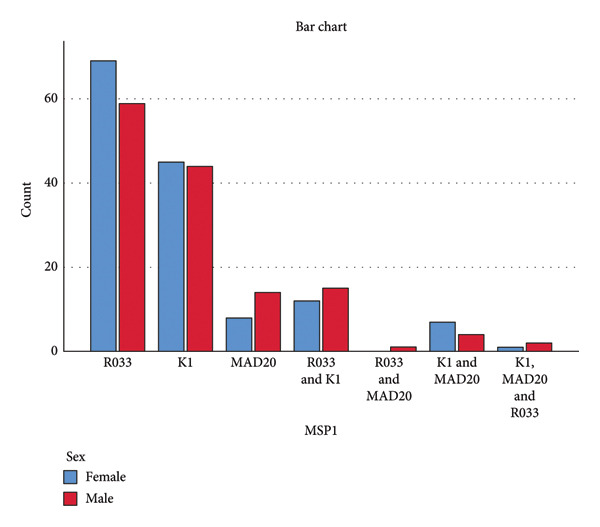
Distribution of Msp1 Block 2 alleles with respect to sex (Pearson *χ* ^2^ = 4.882, *p* = 0.559, linear by linear association *p* = 0.427).

**Figure 10:**
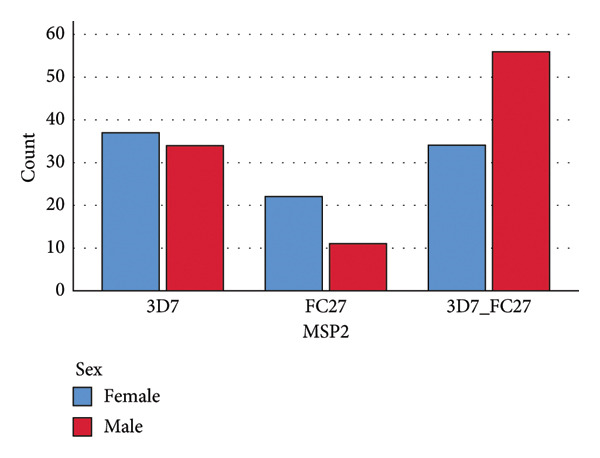
Distribution of Msp2 Block 3 alleles with respect to sex (Pearson *χ* ^2^ = 8.856, *p* = 0.012).

For Msp1, the result suggests that sex has no impact on the distribution of Msp1 Block 2 gene alleles in the study area. No allele was associated with a given sex. In both mono‐ and mixed malaria infections, females and males are equally infected with all the various alleles present in the study area (Figure 9). For Msp2, the result suggests that sex has an impact on the allele distribution of this gene in the study area, with mono‐infection (with either 3D7 or FC27) being more common in females than in males and mixed infection (with 3D7 and FC27) being more common in males than in females (Figure 10).

## 4. Discussion

### 4.1. Genetic Diversity of *P. falciparum* Msp1 and Msp2 Genes in Bamenda

In the field, Msp1 and Msp2 genes have frequently been employed as polymorphic genetic markers to characterize parasite genetic variation [8]. The erythrocyte invasion process of *P. falciparum* involves the Msp1 and Msp2 proteins, which are expressed on the surface of merozoite [23], and are targets of the host immune system [15]. Due to their enormous diversity, Msp1 and Msp2 are essential for identifying subpopulations of *P. falciparum* that differ genetically.

The study’s findings demonstrate the presence of all three *P. falciparum* Msp1 Block 2 gene allelic families in Bamenda (Figure 5). At an allelic frequency of 44.7%, RO33 was the most prevalent allele, followed by K1 with a frequency of 42.6% and MAD20 with a frequency of 12.7%. These findings disagree with earlier research done in Cameroon, where K1 [19] and MAD20 [17, 18] were the predominant alleles. Nonetheless, the findings support those of Ref. [8], done in South West Cameroon in 2020, which found that the RO33 allele was the most prevalent, followed by K1 and MAD20. Due to the balanced selection operating on these two alleles, RO33 and K1 alleles are more prevalent in Bamenda. The genetic drift may be the reason for the low incidence of MAD20 in the study area. Studies published a few years following the introduction of ACTs in Cameroon [17, 18] revealed a significant frequency of the MAD20 allele; it is possible that the use of ACTs contributed to this balancing selection and genetic drift. ACTs influence the selection of *P. falciparum* Msp1 alleles by exerting selection drug pressure on the parasite population. This can lead to shift in the prevalence of specific Msp1 allele [24]. Compared to other allelic types of Msp1, *P. falciparum* with the MAD20 allele may be more susceptible to ACTs. This view is supported by the findings of this study, which demonstrate high genetic diversity within the K1 allelic family with various variants of KI, despite the prolonged use of ACTs in Bamenda. This suggests that *P. falciparum* carrying the KI allele may harbor variants that confer artemisinin resistance. Moreover, *P. falciparum* possessing the RO33 allele may potentially carry an allele for artemisinin resistance because there is only one variant with 180 bp, indicating that this variant is stable in the population. A single nucleotide mutation in the *P. falciparum* DNA template that prevents correct annealing with the primers used to amplify the MAD20 allelic family may also be to blame for the low prevalence of MAD20 observed in the study area.

The genetic diversity of the K1 allelic family is greater than those of MAD20 and RO33, with base pairs (bp) ranging from 100 to 300, and the 200 bp variants being the most prevalent. The genetic diversity of MAD20 was low, with variants ranging from 100 to 200 bp, with the 200 bp variant being the most prevalent. In contrast, RO33 showed no genetic variation with all variants having 180 bp. These findings contradict those of Ref. [8], which found that K1 had variants ranging from 153 to 335 bp, MAD20 had variants ranging from 175 to 205 bp, and RO33 had variants with all being 155 bp. Nonetheless, the outcomes were comparable to those seen in Senegal with K1 variations between 100 and 350 bp, MAD20 variants between 100 and 300 bp, and R033 variants between 160 and 240 bp [24]. These differences in results can be attributed to various transmission dynamics and geographical regions. While this work was conducted in the North West Region of Cameroon, an area that is primarily savanna grassland, high altitude, and low malaria transmission, Refs. [8, 18] were conducted in the South West Region of Cameroon, an area that is primarily rain forest, low altitude, and high malaria transmission. Ref. [17], which demonstrates how altitude and geographic location affect the genetic diversity of *P. falciparum* Msp1 Block 2 genes, supports this viewpoint. The findings of this study and those from Refs. [8, 25] showed that there is no polymorphism within the RO33 allelic family, suggesting that the variant identified may have been fixed in those *P. falciparum* populations by balancing selection.

The results for the *P. falciparum* Msp2 Block 3 genes show that the study region has both alleles for Msp2 Block 3 genes. 3D7 had a 56.7% prevalence rate, while FC27 had a 43.3% prevalence (Figure 6). Mixed infections (3D7 and FC27) were 46.6%, and mono‐infections with 3D7 or FC27 were 36.6% and 17%, respectively. The findings of prior research conducted in the South West Region of Cameroon, where mono‐infections with 3D7 were 4.94%, those with FC27 were 6.51%, and mixed infections with 3D7 and FC27 were 88.7% [8], do not corroborate with the findings of this study. These differences might be as a result of variations in malaria transmission between the two research regions. This study was conducted in Bamenda, Cameroon’s North West Region, which has minimal malaria transmission with 22.4% prevalence [4], as opposed to the South West Region, which has high malaria transmission with a prevalence of 41.5%[4]. According to research, mixed infections are more common in regions where malaria transmission is high and less common in regions where malaria transmission is low [26]. It was impossible to assess the level of genetic diversity of *P. falciparum* Msp2 Block 3 gene before the adoption of ACTs in Cameroon because there were no previous data on this in the study area. As a result, it was challenging to assess the study area’s malaria control measures. A study conducted on Grande Comore Island demonstrates a gradual decline in mixed infections following the introduction of ACTs [27]. Based on this knowledge, it will be essential to carry out a genetic diversity analysis of the *P. falciparum* Msp2 Block 3 gene in the future to evaluate the effectiveness of ACTs in Bamenda.

### 4.2. Association of *P. falciparum* Msp1 and Msp2 Alleles With Malaria Severity in Bamenda

In this research area, the K1 (200 bp) allele was linked to severe malaria (*p* ≤ 0.001), but the R033 allele was linked to a lower risk of severe malaria (Table 4). This outcome is consistent with the findings of Refs. [28, 29], which noted a link between the K1 allele and severe malaria. This conclusion, however, does not support prior discoveries in the South West Region of Cameroon, where K1 was linked to a lower risk of malaria, while MAD20 and RO33 were linked to the increase risk of malaria [19]. These differences might result from different dominant alleles across the two research areas. Study of Ref. [19] was completed at a period when the K1 allele was prevalent in their research area, which suggests that residents of their study area have immunity to the K1 allele, while the current investigation was conducted a few years later and in a different region where RO33 was prominent. This suggests that RO33‐specific immunity exists among individuals in this study area. A previous research conducted in the South West Region of Cameroon, which revealed that RO33 was the dominant allele and was associated with decreased risk of malaria, supports this viewpoint [8]. For Msp2, comparing those with the FC27 allele with those with the 3D7 allele reveals that the former exhibit greater mean parasite density and higher mean temperature; however, the differences were not statistically significant.

### 4.3. Association of *P. falciparum* Msp1 and Msp2 Alleles With Seasons in Bamenda

Compared to the dry season, the study area’s Msp1 Block 2 allele distribution was greater (Figure 7), but the changes were not statistically significant (*p* = 0.518). All of the various alleles were identified both in the wet and dry seasons, demonstrating that no single allele was associated with any particular season. These findings were consistent with Ref. [30], which discovered no correlation between a certain Msp1 Block 2 allele and a specific season. The Msp2 Block 3 allele distribution between the dry and wet seasons was not statistically significant (*p* = 0.447), indicating that no allele was connected to a specific season. Yet, compared to FC27, 3D7 was more prevalent throughout the dry and wet seasons. In the study area, mixed infections (3D7 and FC27) and mono‐infections (3D7 or FC27) were more common during the wet season than during the dry season (Figure 8).

### 4.4. Association of *P. falciparum* Msp1 and Msp2 Alleles With Sex

The Msp1 Block 2 allele distribution between males and females was not statistically significant (*p* = 0.427). No association was found between a particular allele and a specific sex. However, mono‐infection with MAD20 was somewhat more common in males than in females, and mono‐infection with RO33 was slightly more common in females. Males were somewhat more likely than females to have mixed infections (Figure 9). These outcomes resemble those of Ref. [30]. The results for Msp2 Block 3 alleles showed that the allele distributions between males and females were statistically significant (*p* = 0.012). This shows that there is a relationship between sex and the Msp2 Block 3 alleles, with the alleles preferring to infect a certain sex more in both mono‐infections and mixed infections. Females were more likely than males to get mono‐infection with 3D7 or FC27, but males were more likely to contract mixed infection with 3D7 and FC27 (Figure 10). Females clear mono‐infections more slowly than males do; this might explain why females have a higher rate of mono‐infections. According to a study, mono‐infections healed more quickly than mixed infections [31]. Th1 responses inhibit parasite growth, which is thought to be the primary mechanism mediating reduced susceptibility to infection in males. Males develop higher Th1 responses, including elevated IFN synthesis, whereas females exhibit higher IL‐10 production during the early phase of parasitic infections [32]. The reduced prevalence of mono‐infections in males shows that mono‐infections with 3D7 or FC27 clear more quickly than mixed infections with 3D7 and FC27 during malaria infections. However, given the paucity of available information, this idea requires additional research. It is possible that females resolve mixed infections more quickly than males do, explaining why males have a greater rate of mixed infections than females do. Mixed infection requires more time to heal; hence, antimalarial antibodies are needed. There have been reports of more antimalarial antibodies in females than in males [33]. Another research found that females had a lower prevalence of *P. falciparum* infections than males did, but this difference did not appear to be attributable to a lower incidence of infections but rather to a quicker rate of mixed infection clearance [31]. The effect of steroid hormones may be the cause of the differential in the clearance of mixed infections between males and females. Males may be more susceptible to *P. falciparum* mixed infections than females due to the immunomodulatory effects of testosterone [32]. According to a study done on mice, male mice have a greater death rate as well as more severe weight loss, anemia, and parasitemia than female mice. The research also revealed that castration of male mice increases their resistance to *Plasmodium* infections, but testosterone therapy diminishes resistance to *Plasmodium* infections in female mice [34]. Another study suggested that estrogen may shield females who are infected with malaria from developing illness symptoms [35].

## 5. Conclusion

The genetic diversity of the *P. falciparum* Msp1 and Msp2 genes in Bamenda was first described in this work. In Bamenda, the most common allele for the Msp1 Block 2 gene was RO33, whereas the most common allele for the Msp2 Block 3 gene was 3D7. This study demonstrates a link between severe malaria and the K1 allele of *P. falciparum* Msp1 in Bamenda. It also offers the first details on the relationship between the Msp2 alleles of *P. falciparum* and sex. These results would be useful in assessing the efficacy of malaria prevention measures implemented in Bamenda as well as in selecting and designing malaria prevention measures that are suitable for implementation in Bamenda, North West Region of Cameroon.

## Ethics Statement

The study received ethical approval from the Institutional Review Board of the Faculty of Health Science, University of Buea (Reference No. 2019/872‐11/UB/SG/IRB/FHS). Administrative authorization was obtained from the North West Regional Delegation of Public Health, Cameroon (Reference No. 79/L/NWR/RDPH). Only children who fulfilled the specific inclusion criteria and assent given by their parents or guardians after adequate sensitization on the research objectives, possible risks, and benefits were enrolled into the study.

## Consent

The authors have nothing to report.

## Disclosure

All authors read and approved the final copy of the manuscript.

## Conflicts of Interest

The authors declare no conflicts of interest.

## Author Contributions

Che Roland Achungu, Damian Nota Anong, and Kamena Faustin conceived and designed the experiments; Che Roland Achungu carried out the experiment and produced the first draft of the manuscripts; Che Roland Achungu and Robert Adamu Shey performed molecular analysis of the data; and Che Roland Achungu, Cevie Jesenta Tabe, and Chi Achille Djouosseu participated in sample collections and genotyping.

## Funding

This study did not receive any funding in any form.

## Data Availability

All data generated or analyzed during this study are included in this manuscript.
